# Early Class III treatment with Hybrid-Hyrax -Facemask in comparison to Hybrid-Hyrax-Mentoplate – skeletal and dental outcomes

**DOI:** 10.1186/s40510-018-0239-8

**Published:** 2018-10-22

**Authors:** Jan H. Willmann, Manuel Nienkemper, Nour Eldin Tarraf, Benedict Wilmes, Dieter Drescher

**Affiliations:** 1 Department of Orthodontics, Heinrich-Heine-University, Kasernenstr. 1, 40213 Düsseldorf, Germany; 2 Sydney, Australia; 3 Private Practice, Düsseldorf, Germany

**Keywords:** Class III, Facemask, Mini-plates, Skeletal anchorage, Rapid maxillary expansion

## Abstract

**Background:**

Protraction of maxilla is usually the preferred and more commonly used treatment approach for skeletal Class III with a retrognathic maxilla. The aim of this study was the comparison of the skeletal and dental effects of two skeletally borne appliances for maxillary protraction: a) Hybrid-Hyrax in combination with facemask (FM), b) Hybrid-Hyrax in combination with Mentoplate (ME).

**Methods:**

Thirty four Patients (17 facemask, 17 Mentoplate) were investigated by means of pre- and posttreatment cephalograms. The two groups matched with regard to treatment time, age gender and type of dentoskeletal deformity before treatment.

**Results:**

Both groups showed a significant forward movement of A-point (FM GROUP: SNA + 2.23° ± 1.30°— *p* 0.000*; ME: 2.23° ± 1.43°— *p* 0.000*). B-Point showed a larger sagittal change in the FM Group (SNB 1.51° ± 1.1°— *p* 0.000*) compared to the ME group (SNB: − 0.30° ± 0.9°— *p* 0.070). The FM group showed a significant increase of the ML-NL + 1.86° ± 1.65° (*p* 0.000*) and NSL-ML + 1.17° ± 1.48 (*p* 0.006*). Upper Incisor inclination did not change significantly during treatment in both groups as well as the distance of the first upper Molar in relation to A-point.

**Conclusion:**

Both treatments achieve comparable rates of maxillary protraction, without dentoalveolar side effects. Skeletal anchorage with symphysial plates in the mandible provides greater vertical control and might be the treatment of choice in high angle patients.

## Background

Morphological features of skeletal class III malocclusion may comprise mandibular prognathism, maxillary retrognathism or a combination of both. Cross-sectional studies revealed a prevalence of class III patients with a retrusive maxilla between 32 and 63%, depending on the investigated population, ethnicity, and sex [1–3]. In these patients, protraction of the deficient maxilla represents a causal treatment approach [3–11].

Sagittal orthopaedic forces to protract the maxillary complex were commonly applied to the upper dental arch [6, 12, 13]. This approach incurred well-known side effects such as proclination of the upper front teeth, bite opening, mesial movement of the lateral segments, and constriction of unerupted canines [14–18].

New skeletal anchorage concepts involving surgical mini-plates or mini-implants have been developed to address these problems [19–21]. Directing orthopaedic forces directly into the bony structures of the midface promised a significant reduction of dental side effects as well as an enhancement of skeletal response. To further increase orthopaedic treatment effects, some maxillary protraction protocols include rapid maxillary expansion (RME) in order to stimulate the midface sutures [12, 18, 22]. Interestingly, systematic reviews and meta-analyses representing a high level of evidence either advocate or dismiss the positive effect of RME [10, 23–25]. RME can be carried out purely bone-borne or with a combination of dental and skeletal anchorage using mini-implants in the anterior palate (Hybrid-Hyrax).

Traditionally, maxillary protraction has been performed by extraoral traction using various types of facemasks [26, 27]. The associated skeletal treatment effects have been documented extensively in numerous clinical studies: advancement and anterior rotation of the maxilla, sagittal growth inhibition and posterior rotation of the mandible, and increase of the vertical dimension [28–30].

As an alternative, skeletal anchorage in the lower jaw eliminates the need for extraoral devices, which might have a positive effect on patient’s acceptance and compliance. The Mentoplate, which was used for maxillary protraction in one study group, is inserted subapical to the lower incisors and can be inserted prior to canine eruption [31].

**Fig. 1:**
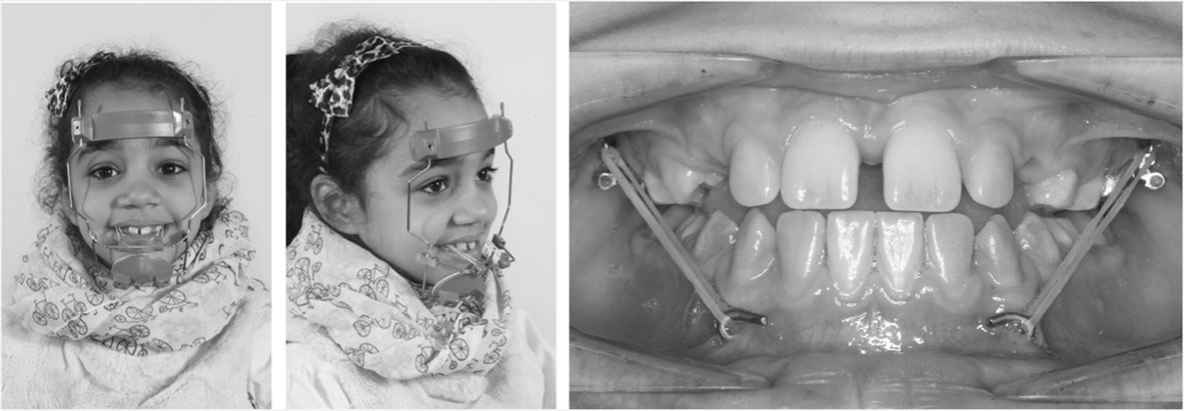
Exemplary presentation of a patient wearing a facemask (left) and a Mentoplate (right)

The aim of this retrospective study was to investigate the skeletal and dental effects of two skeletally borne appliances for maxillary protraction: (a) Hybrid-Hyrax in combination with facemask (FM) and (b) Hybrid-Hyrax in combination with Mentoplate (ME) (Fig. 1). The null hypothesis was that there is no difference regarding the skeletal and dental effects between the different treatment modalities.

## Methods

Initially, a group of 50 consecutively treated patients was considered for this study.

Inclusion criteria were as follows:

- Moderate/severe class III: WITS ≤ − 2.0 mm
- Age ≥ 7 years to ≤ 12 years
- Treated according to a standardised protocol (see below)
- Lateral cephalograms before and after treatment
- Anterior crossbite or incisor edge-to-edge relationship, class III molar relationship

Exclusion criteria were as follows:

- Craniofacial anomalies
- Systemic diseases
- Forced or functional bite

Thirty-four patients (17 facemask, 17 Mentoplate) fulfilled the inclusion/exclusion criteria. The group compositions can be found in Tables 1 and 2.

**Table 1:** Group allocation

	Male	Female	Total
Facemask	8	9	17
Mentoplate	7	10	17
Total	15	19	34

Chi-square 0.500 n.s

**Table 2:** Age distribution

	Age
Facemask	8.74 ± 1.20
Mentoplate	9.43 ± 0.95
*T* test	0.072 n.s.

### Treatment protocol

**Fig. 2:**
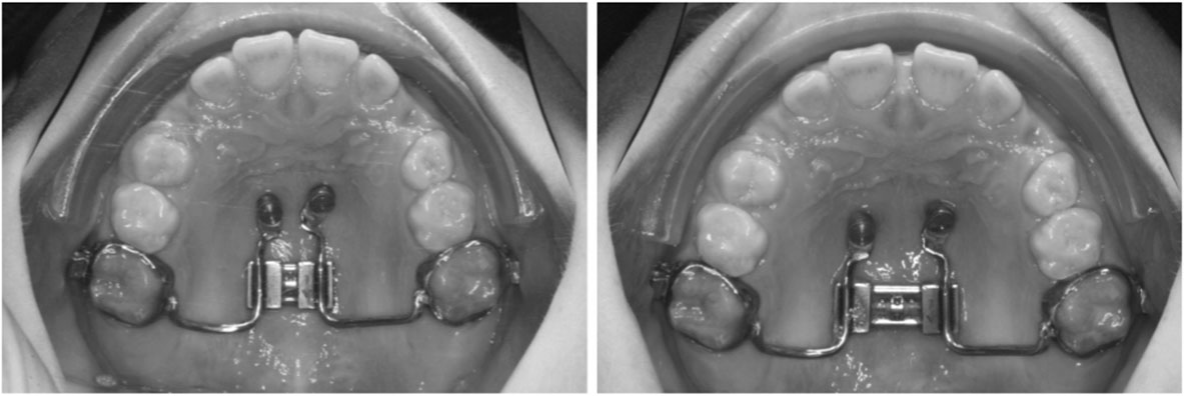
Hybrid-Hyrax- before and after maxillary expansion

A Hybrid-Hyrax device fitted on two paramedian mini-implants in the anterior palate (2 × 9 mm, Benefit, PSM, Tuttlingen, Germany) for RME was used in all patients. RME was performed activating the Hyrax screw by 90° turns four times a day, resulting in an expansion of 0.8 mm per day (Fig. 2).

The Mentoplate (PSM, Tuttlingen, Germany) was surgically inserted at the department for oral surgery under local anaesthesia 2 weeks prior to RME. Protraction was started simultaneously with RME in both groups.

**Fig. 3:**
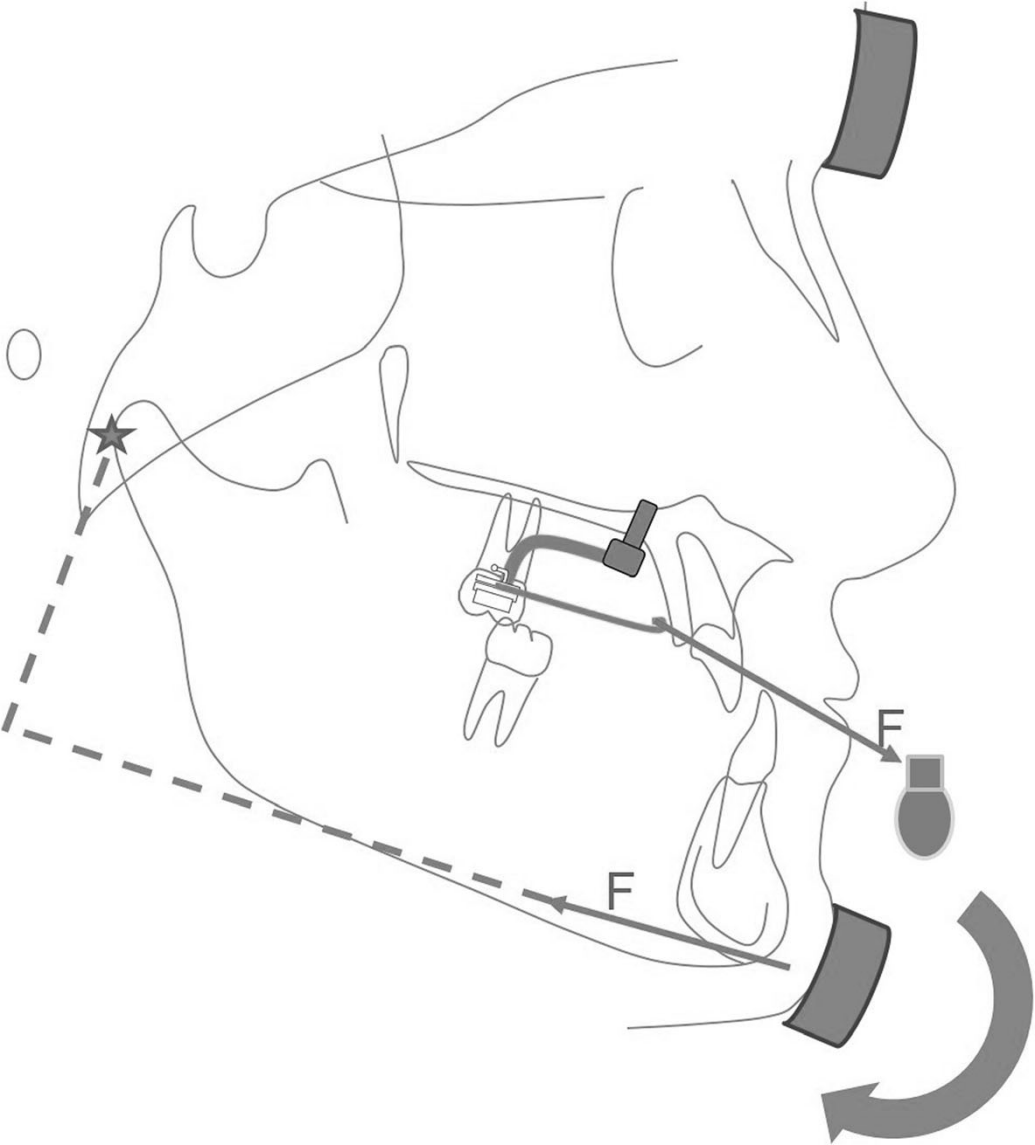
Schematic diagram of the facemask

**Fig. 4:**
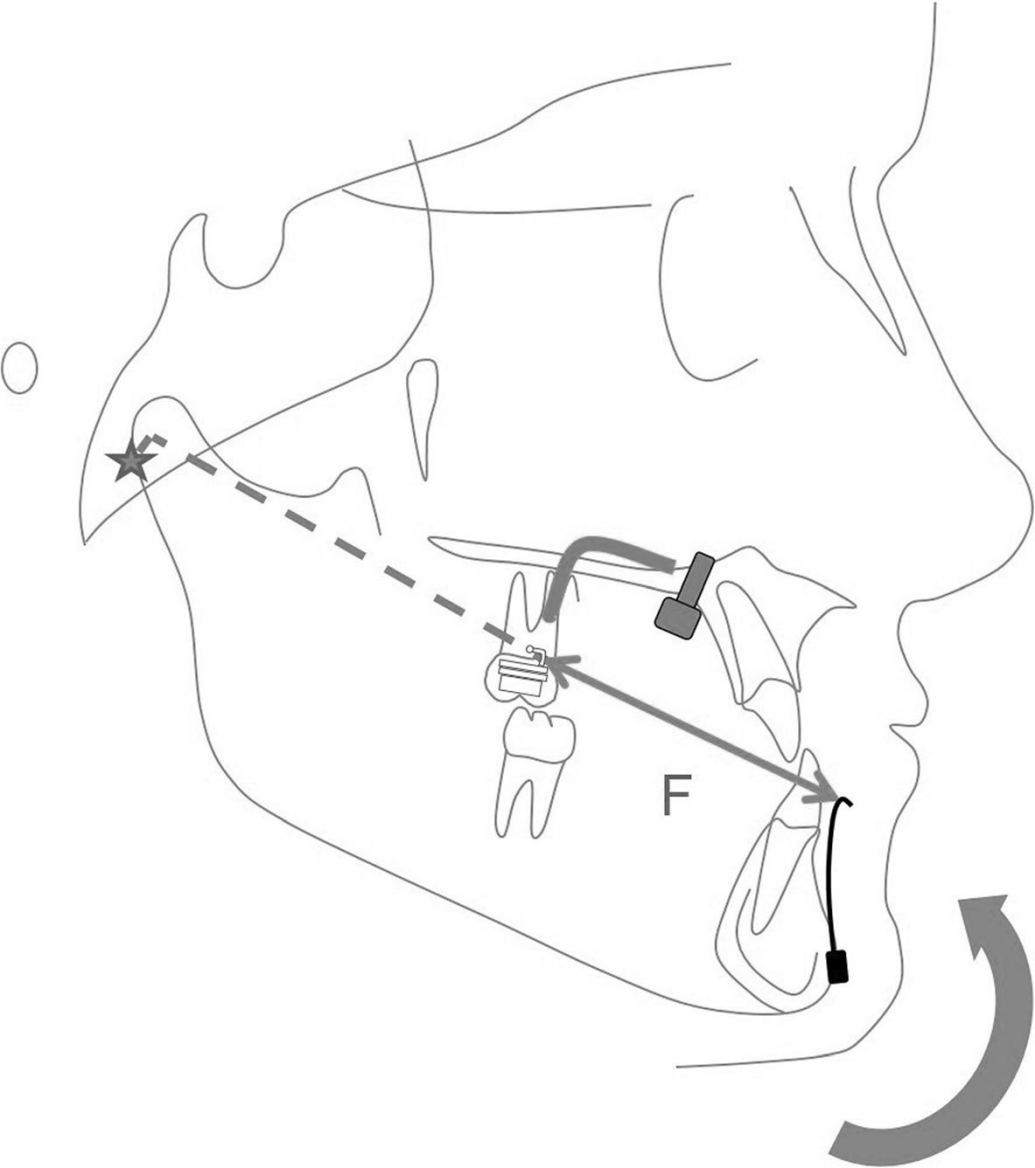
Schematic diagram of the Mentoplate

The FM group was instructed to wear 400 g elastics on each side for 14–16 h per day [6, 11, 32]. The force vector of the elastics, between the FM and the Hybrid-Hyrax, was adjusted to have an inclination of 20–30° relative to the occlusal plane (Fig. 3). The ME group was instructed to wear 200 g elastics on each side, between the Hybrid-Hyrax and the Mentoplate, for 24 h per day. Cl. III elastics were worn with an inclination of 10–15° relative to the occlusal plane (Fig. 4).

### Cephalometric analysis

Digital pre- (T0) and posttreatment cephalograms (T1) (Sirona Orthopos XG plus; Bensheim, Germany) were calibrated and analysed. Measurements and superimpositions according to stable cranial structures the anterior border of Sella and median border of the orbital roof were performed by the same operator using the Software ImageCollector. Blinding of the operator was only possible for the pre-treatment cephalograms, since the Mentoplate was still in place in all of the post-treatment radiographs.

**Fig. 5:**
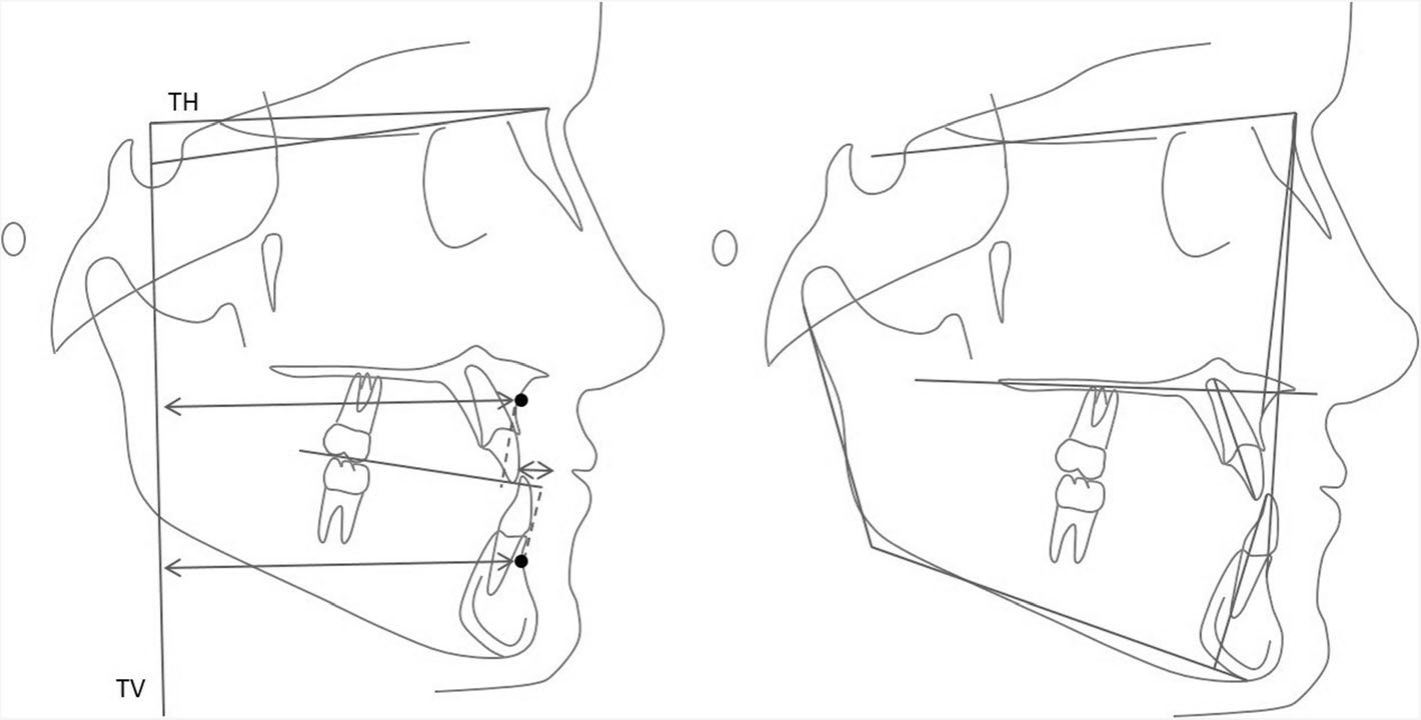
Cephalometric analysis, left sagittal linear measurements (TH—true horizontal; TV—true vertical); right angular measurements

Cephalometric landmarks and planes and their definitions are presented in Fig. 5 and Table 3. Fifteen randomly selected cephalograms were retraced on two different occasions within a 2-week interval by one examiner. The intraclass correlation coefficient (ICC) ranged between 0.93 and 0.98.

**Table 3:** Cephalometric values; comparison of initial values before treatment

Variables	Facemask T0	Mentoplate T0	*p* values	CI 95%	
SNA°	79.41 ± 2.86	79.23 ± 3.08	0.865	− 1.90	2.26
SNB°	80.51 ± 3.26	80.09 ± 3.05	0.703	− 1.79	2.63
ANB°	− 1.10 ± 1.98	− 0.86 ± 1.83	0.714	− 1.58	1.09
WITS mm	− 5.39 ± 1.47	− 5.83 ± 1.35	0.369	− 0.55	1.43
ATV mm	59.99 ± 2.99	59.42 ± 4.97	0.685	− 2.29	3.45
BTV mm	59.32 ± 4.78	58.24 ± 7.04	0.604	− 3.13	5.29
ABTV mm	0.67 ± 3.16	1.18 ± 2.88	0.629	− 2.62	1.61
ML-NL°	26.06 ± 5.44	27.87 ± 6.18	0.371	− 5.88	2.26
ML-NSL°	32.65 ± 6.27	34.95 ± 6.94	0.317	− 6.93	2.32
NSL-NL°	6.58 ± 2.93	7.08 ± 3.74	0.812(MWU)	− 2,85	1,85
AR-GO-ME°	126.92 ± 7.05	128.10 ± 4.99	0.643	− 6.23	3.95
MOK-A mm	26.37 ± 2.21	26.43 ± 1.93	0.929	− 1.51	1.39
U1-PP°	108.19 ± 7.85	110.36 ± 6.79	0.396	− 7.30	2.96
L1-ML°	86.87 ± 6.27	86.52 ± 6.93	0.892(MWU)	− 4,27	4,97

*MWU* Mann-Whitney *U* test

### Statistics

Statistical analysis was carried out using SPSS (IBM, Version 23). Measurements were tested for normal distribution using the Shapiro-Wilk test. Depending on these tests, statistical comparison of mean values was carried out using parametric or non-parametric tests, respectively. Intra-group differences were identified using Student’s *t* test for dependent samples or Wilcoxon test. Differences between the groups were tested using *t* test for independent samples or Mann-Whitney *U* test. The confidence interval was set to 95%.

## Results

Treatment time, age and gender distribution did not show significant differences between the groups. (Tables 1, 2 and 4). Initial cephalometric values revealed did not differ significantly at T0 (Table 3).

**Table 4:** Treatment time

	Treatment time in years
Facemask	0.79 ± 0.26
Mentoplate	0.87 ± 0.25
*T* test	0.362 n.s.

The skeletal effects for each group are shown in Tables 5 and 6. The differences between the groups are shown in Table 7.

**Table 5:** Skeletal and dental treatment effects in the facemask group

Variables	Facemask T0	Facemask T1	*p* values	CI 95%	
SNA°	79.41 ± 2.86	81.66 ± 2.92	0.000^*^	1.60	2.90
SNB°	80.51 ± 3.26	79.02 ± 3.27	0.000^*^	− 2.06	− 0.91
ANB°	− 1.10 ± 1.98	2.65 ± 2.34	0.000^*^	3.00	4.49
WITS mm	− 5.39 ± 1.47	− 0.57 ± 1.51	0.000^*^	4.02	5.52
ATV mm	59.99 ± 2.99	62.42 ± 3.47	0.000^*^	1.71	3.13
BTV mm	59.32 ± 4.78	57.52 ± 5.05	0.002^*^	− 2.81	− 0.74
ABTV mm	0.67 ± 3.16	4.90 ± 3.60	0.000^*^	3.30	5.14
ML-NL°	26.06 ± 5.44	27.95 ± 6.12	0.000^*^	1.03	2.74
ML-NSL°	32.65 ± 6.27	33.79 ± 6.11	0.149	0.37	1.90
NSL-NL°	6.58 ± 2.93	5.84 ± 3.96	0.148 (W)	− 0.27	1.76
AR-GO-ME°	126.92 ± 7.05	127.33 ± 6.92	0.001^*^	0.19	0.61
MOK-A mm	26.37 ± 2.21	26.30 ± 2.19	0.246	− 0.05	0.19
U1-PP°	108.19 ± 7.85	107.04 ± 6.92	0.473	− 2.16	4.47
L1-ML°	86.87 ± 6.27	83.04 ± 4.26	0.028^*^ (W)	− 6.99	− 0.67

*W* Wilcoxon

*significant at *p* < 0.05

**Table 6:** Skeletal and dental treatment effects in the Mentoplate group

Variables	Mentoplate T0	Mentoplate T1	*p* values	CI 95%	
SNA°	79.23 ± 3.08	81.47 ± 3.15	0.000*	1.49	2.97
SNB°	80.09 ± 3.05	79.79 ± 3.20	0.070	− 0.42	0.97
ANB°	− 0.86 ± 1.83	1.68 ± 1.55	0.000*	2.20	3.20
WITS mm	− 5.83 ± 1.35	− 1.69 ± 1.32	0.000*	3.74	5.05
ATV mm	59.42 ± 4.97	62.09 ± 5.03	0.000*	1.90	3.44
BTV mm	58.24 ± 7.04	58.50 ± 7.24	0.973	− 0.91	0.88
ABTV mm	1.18 ± 2.88	3.59 ± 2.91	0.000*	2.16	3.17
ML-NL°	27.87 ± 6.18	27.97 ± 6.05	0.869	− 1.26	1.07
ML-NSL°	34.95 ± 6.94	34.40 ± 6.87	0.055	− 0.01	1.11
NSL-NL°	7.08 ± 3.74	6.44 ± 3.56	0.229	− 0.44	1.73
AR-GO-ME°	128.10 ± 4.99	125.14 ± 8.36	0.000*	− 3.96	− 1.94
MOK-A mm	26.43 ± 1.93	26.32 ± 1.86	0.054	0.00	0.22
U1-PP°	110.36 ± 6.79	110.78 ± 5.12	0.752	− 3.22	2.37
L1-ML°	86.52 ± 6.93	85.97 ± 6.22	0.556	− 1.41	2.52

*significant at *p* < 0.05

**Table 7:** Group differences between the facemask and Mentoplate group

Variables	Facemask (ΔT0 − T1)	Mentoplate (ΔT0 − T1)	*p* values	CI 95%	
SNA°	2.23 ± 1.30	2.23 ± 1.43	0.995	− 0.96	0.95
SNB°	− 1.51 ± 1,13	− 0.30 ± 0.98	0.002*	− 1.95	− 0.47
ANB°	3.75 ± 1.45	2.54 ± 0.99	0.008*	0.33	2.08
WITS mm	4.81 ± 1,38	4.14 ± 1.25	0.147	− 0.25	1.59
ATV mm	2.38 ± 1,42	2.67 ± 1.49	0.557	− 1.31	0.72
BTV mm	− 1.87 ± 2,08	0.26 ± 1.75	0.003*	− 3.47	− 0.78
ABTV mm	4.24 ± 1.78	2.41 ± 0.99	0.001*	0.82	2.84
ML-NL°	1.89 ± 1.65	0.12 ± 2.11	0.005*	0.61	3.27
ML-NSL°	1.17 ± 1.48	− 0.55 ± 1.09	0.001*	0.80	2.63
NSL-NL°	− 0.72 ± 1.99	− 0.49 ± 2.06	0.501 (MWU)	− 1.64	1.19
AR-GO-ME°	0.40 ± 0.41	− 2.96 ± 1.96	0.000*	2.36	4.35
MOK-A mm	− 0.07 ± 0.24	− 0.11 ± 0.22	0.624	− 0.12	0.20
U1-PP°	− 1.15 ± 6.45	0.57 ± 5.49	0.407	− 5.91	2.46
L1-ML°	− 3.84 ± 6.13	− 0.56 ± 3.83	0.081 (MWU)	− 6.85	0.29

*MWU* Mann-Whitney *U* test

*significant at *p* < 0.05

Anterior and posterior crossbites were corrected in all individuals. Neither implant or plate failures nor breakages of the appliances occurred.

## Discussion

The main goal of early class III treatment of patients with maxillary retrognathia is to achieve maxillary protraction and growth restriction of the mandible without undesirable side effects such as mesial migration of the upper dentition and vertical skeletal changes.

Various different strategies exist to achieve these objectives:

- The BAMP (Bone anchored Maxillary Protraction) protocol [19]
- The Miniscrew Implants/Facemask combination [33, 34]
- Two miniplates laterally to the aperture piriformis in conjunction with a facemask [35]
- The Hybrid-Hyrax Facemask/Mentoplate combination [31, 36]

which was examined in this retrospective study. The groups were comparable regarding their skeletal pattern, age, sex, and treatment time. The review of the confidence interval show, that a sufficient number of patients were evaluated. The significant differences are thereby supported by alpha and beta errors.

Maxillary protraction was carried out successfully in both groups, leading to a significant improvement of the maxillary position. In both groups, similar changes were induced regarding the SNA-Angle during a comparable treatment period (SNA + 2.23°), and a significant improvement of the WITS-appraisal (FM Group 4.81 mm, ME 4.14 mm) was found. These changes comply with the reported treatment effects on SNA with range of 1–3° achieved by maxillary protraction [8–10, 37]. The values we found are slightly higher than those of conventional RME and FM therapy. In a controlled clinical study, Westwood et al. found increases of 1.6° in SNA and 4.3 mm in the Wits appraisal [7]. A meta-analysis of conventional maxillary protraction reported a mean increase of SNA by 1.4° [24].

Many clinicians favour the use of RME to open the midface sutures to improve the skeletal effect. The RME/FM protocol demonstrates superior maxillary protraction when performed in the early mixed dentition [6, 18]. Consequently, the timing of treatment seems to be of paramount importance. Current evidence seems to be slightly in favour to combine RME and maxillary protraction during early Class III treatment, which gave reason to perform RME in all patients included in this study [24].

Mini-plate anchored maxillary protraction as described by de Clerck showed good skeletal effects in the late mixed or early permanent dentition [38, 39]. Since the Mentoplate is inserted in the subapical region of the lower incisors, awaiting the eruption of the lower canines is not necessary, allowing for an earlier onset of treatment [31, 40]. Currently, it is not very clear whether early and late onset of treatment using purely bone-borne protraction devices is more effective.

The skeletal effect in the maxilla seems to be improved, if the orthopaedic forces are applied directly to the maxillary bone with the help of skeletal anchorage instead of using tooth-borne appliances [39, 41, 42]; also, a reduction of dental side effects can be observed. The usual side effects occurring during protraction with tooth-borne appliances such as proclination of the incisors, space loss for the canines and mesial migration of the molars could not be observed in both study groups. Therefore, the majority of the overjet correction (FM group 3.51 mm, ME group 3.06 mm) was due to favourable skeletal changes rather than dentoalveolar compensation.

For protraction of the maxilla, heavy forces of 400 up to 1500 g are recommended with FM therapy, to facilitate a sufficient orthopaedic effect [43]. For purely bone-anchored protraction protocols, lighter forces are recommended. De Clerck used an initial force of 100 g, which is gradually increased to 250 g, with a recommended full time wear of the Cl. III elastics. In this study, 200 g were used over the whole treatment time. Intraoral elastics can be worn full time without affecting the patient facial appearance, which might be a key to increase patient’s compliance. Subjective wear time analysis revealed a FM wear time of 14 h per day [14, 18, 44, 45]. In a case study, an objective wear time measurement showed an average wear time of 9 h a day [46]. Apparently, the recommended heavy forces in conventional appliances stem from the limited wear times of these extraoral devices. In contrast, purely intraoral skeletally anchored devices can be worn over a longer period of time during a day, thus producing a comparable skeletal effect at lower force levels. As in all other studies, it would have been most desirable being able to objectively measure the exact wear times of the elastics for maxillary protraction.

As mentioned above, the skeletal effects found in the FM and ME groups on the maxilla where comparable. This was not true for the mandible where a significant decrease of the SNB angle was found. Analysis of the vertical cephalometric measurements revealed a significant opening of the interbase angle (ML-NL) in the FM group which was mainly caused by a posterior rotation of the mandible. In other words, B point effectively moved down and backwards in the FM-group, which might be due to the chincap effect of the facemask [4]. Consequently, the skeletal effect on the mandible in the FM-group is more of a vertical nature, described by a posterior rotation of the mandible (Fig. 3). In contrast, the B-point remains stable in the ME-group (Fig. 4). These findings were consistent with those of Cevidanes and other authors, who reported a greater vertical control and less opening rotation of the mandible when applying forces to symphyseal plates [38, 39, 47]. The gonial angle decreased significantly in the ME-group, which might be due to changes in the direction of condylar and ramus growth [48].

The results represent short-term observation within the limitations of a retrospective study. Further observation of these patients would be desirable to be able to draw long-term conclusions of these treatment modalities.

## Conclusions

Both treatment options achieve comparable rates of maxillary protraction, without dentoalveolar side effects. The Mentoplate can be inserted before eruption of the mandibular canines allowing an early onset of class III treatment. The need to wear a facemask is eliminated. Hence, it can be alternative if patients refuse to wear a facemask. Skeletal anchorage with symphyseal plates in the mandible provides greater vertical control and might be the treatment of choice in high angle patients.
